# TCRD and Pharos 2021: mining the human proteome for disease biology

**DOI:** 10.1093/nar/gkaa993

**Published:** 2020-11-06

**Authors:** Timothy K Sheils, Stephen L Mathias, Keith J Kelleher, Vishal B Siramshetty, Dac-Trung Nguyen, Cristian G Bologa, Lars Juhl Jensen, Dušica Vidović, Amar Koleti, Stephan C Schürer, Anna Waller, Jeremy J Yang, Jayme Holmes, Giovanni Bocci, Noel Southall, Poorva Dharkar, Ewy Mathé, Anton Simeonov, Tudor I Oprea

**Affiliations:** National Center for Advancing Translational Science, 9800 Medical Center Drive, Rockville, MD 20850, USA; Translational Informatics Division, Department of Internal Medicine, University of New Mexico Health Sciences Center, Albuquerque, NM 87131, USA; National Center for Advancing Translational Science, 9800 Medical Center Drive, Rockville, MD 20850, USA; National Center for Advancing Translational Science, 9800 Medical Center Drive, Rockville, MD 20850, USA; National Center for Advancing Translational Science, 9800 Medical Center Drive, Rockville, MD 20850, USA; Translational Informatics Division, Department of Internal Medicine, University of New Mexico Health Sciences Center, Albuquerque, NM 87131, USA; Novo Nordisk Foundation Center for Protein Research, Faculty of Health and Medical Sciences, University of Copenhagen, 2200 Copenhagen, Denmark; Institute for Data Science and Computing, University of Miami, Coral Gables, FL 33146, USA; Department of Molecular and Cellular Pharmacology, Miller School of Medicine, University of Miami, Miami, FL 33136, USA; Institute for Data Science and Computing, University of Miami, Coral Gables, FL 33146, USA; Institute for Data Science and Computing, University of Miami, Coral Gables, FL 33146, USA; Department of Molecular and Cellular Pharmacology, Miller School of Medicine, University of Miami, Miami, FL 33136, USA; Sylvester Comprehensive Cancer Center, Miller School of Medicine, University of Miami, Miami, FL 33136, USA; UNM Center for Molecular Discovery, University of New Mexico Health Sciences Center, Albuquerque, NM 87131, USA; Translational Informatics Division, Department of Internal Medicine, University of New Mexico Health Sciences Center, Albuquerque, NM 87131, USA; Translational Informatics Division, Department of Internal Medicine, University of New Mexico Health Sciences Center, Albuquerque, NM 87131, USA; Translational Informatics Division, Department of Internal Medicine, University of New Mexico Health Sciences Center, Albuquerque, NM 87131, USA; National Center for Advancing Translational Science, 9800 Medical Center Drive, Rockville, MD 20850, USA; National Center for Advancing Translational Science, 9800 Medical Center Drive, Rockville, MD 20850, USA; National Center for Advancing Translational Science, 9800 Medical Center Drive, Rockville, MD 20850, USA; National Center for Advancing Translational Science, 9800 Medical Center Drive, Rockville, MD 20850, USA; Translational Informatics Division, Department of Internal Medicine, University of New Mexico Health Sciences Center, Albuquerque, NM 87131, USA; Novo Nordisk Foundation Center for Protein Research, Faculty of Health and Medical Sciences, University of Copenhagen, 2200 Copenhagen, Denmark; UNM Comprehensive Cancer Center, University of New Mexico Health Sciences Center, Albuquerque, NM 87131, USA; Department of Rheumatology and Inflammation Research, Institute of Medicine, Sahlgrenska Academy at University of Gothenburg, 40530 Gothenburg, Sweden

## Abstract

In 2014, the National Institutes of Health (NIH) initiated the Illuminating the Druggable Genome (IDG) program to identify and improve our understanding of poorly characterized proteins that can potentially be modulated using small molecules or biologics. Two resources produced from these efforts are: The Target Central Resource Database (TCRD) (http://juniper.health.unm.edu/tcrd/) and Pharos (https://pharos.nih.gov/), a web interface to browse the TCRD. The ultimate goal of these resources is to highlight and facilitate research into currently understudied proteins, by aggregating a multitude of data sources, and ranking targets based on the amount of data available, and presenting data in machine learning ready format. Since the 2017 release, both TCRD and Pharos have produced two major releases, which have incorporated or expanded an additional 25 data sources. Recently incorporated data types include human and viral-human protein–protein interactions, protein–disease and protein–phenotype associations, and drug-induced gene signatures, among others. These aggregated data have enabled us to generate new visualizations and content sections in Pharos, in order to empower users to find new areas of study in the druggable genome.

## INTRODUCTION

It is widely acknowledged that only a subset of the human genome that is considered ‘druggable’ is subject to scientific inquiry (1). Since 2014, the National Institutes of Health (NIH) initiative Illuminating the Druggable Genome (IDG), has been working toward the goal of shedding light on understudied protein targets that could be potentially modulated by small molecules or biologics. Pharos and the Target Central Resource Database (TCRD) are open-access resources developed as a part of the IDG program and jointly serve as the knowledge hub for over 20 000 human protein targets (2,3). First introduced and published in the 2017 NAR database issue (4), TCRD collates information from several gene/protein data sources and Pharos serves as a web interface that presents this information to users. Since its initial launch, the Pharos paper was cited in more than 110 times *cf*. Google Scholar, and the portal is accessed on average by ∼1600 new visitors monthly, with a total of ∼ 425 000 pageviews and >20 000 full TCRD database downloads (as of 3 September 2020). TCRD has been enhanced by inclusion of new data types and data from emerging resources, which were prepared for machine-learning readiness. The scope of TCRD has also been expanded, moving past the initial area of focus of the druggable genome to aggregate data about the entire human proteome. The first published version of Pharos was based on TCRD version 3.0, the latest version uses TCRD version 6.7, which currently aggregates data from 78 data sources (see Supplementary Information). Furthermore, the search functionality on the Pharos web server has been upgraded to use the graph querying language (GraphQL; https://graphql.org/) API that facilitates faster data retrieval, directly from TCRD.

In the current paper, we describe changes implemented for the 2021 version, such as new data sources and how data from these sources have been integrated into TCRD and presented in Pharos. The latest architecture of the database and both new and improved features implemented in the Pharos platform are described in the following sections of the paper.

## MATERIALS AND METHODS

The newly added data includes mouse and rat proteins from UniProt (5), with their associated phenotype data extracted from the International Mouse Phenotyping Consortium (6) and the Rat Genome Database (7), respectively. The Disease Ontology (DO) (8) data were further extended and newer ontologies such as Rat Disease Ontology (9) and Mammalian Phenotype Ontology (10) were added to facilitate comparison of target-disease and target-phenotype associations across multiple species. Similarly, additional data was included from GWAS (11) and OMIM (https://omim.org/) resources. The ‘Disease details’ page reflects these changes in addition to the other improvements made in displaying the list of associated targets. Throughout this manuscript, the term ‘target’ refers to ‘gene or protein of interest’, as sometimes attributes are related to genes (e.g. orthologs), and sometimes to proteins. However, TCRD is based on ‘reviewed’ (manually curated) human protein entries from UniProt.

Target expression data was primarily extracted from GTEx (12), the Human Protein Atlas (HPA) (13), UniProt and TISSUES (14). The GTEx dataset was further extended to include sex-specific expression values. In addition, cell line expression data was added from HPA and the Human Proteome Map (15) and expression data was collected for orthologous genes from 17 different species. Other expression datasets integrated into the latest version are the Cancer Cell Line Encyclopedia (16) and cell perturbation expression data from the Library of Integrated Network-Based Cellular Signatures (17). Furthermore, the target expression panel was visually upgraded with interactive anatomograms (18) (https://www.ebi.ac.uk/gxa) for both sexes which further provides systematic mappings to the source tissues.

Protein–protein interaction (PPI) is another data type that was included in the latest version by adding the most recent PPI data from STRING 11 (19). Given the COVID-19 pandemic, viral-human PPI data were added from P-HIPSTer (20) to help explore PPIs between viral pathogens and human proteins.

**Figure 1.**
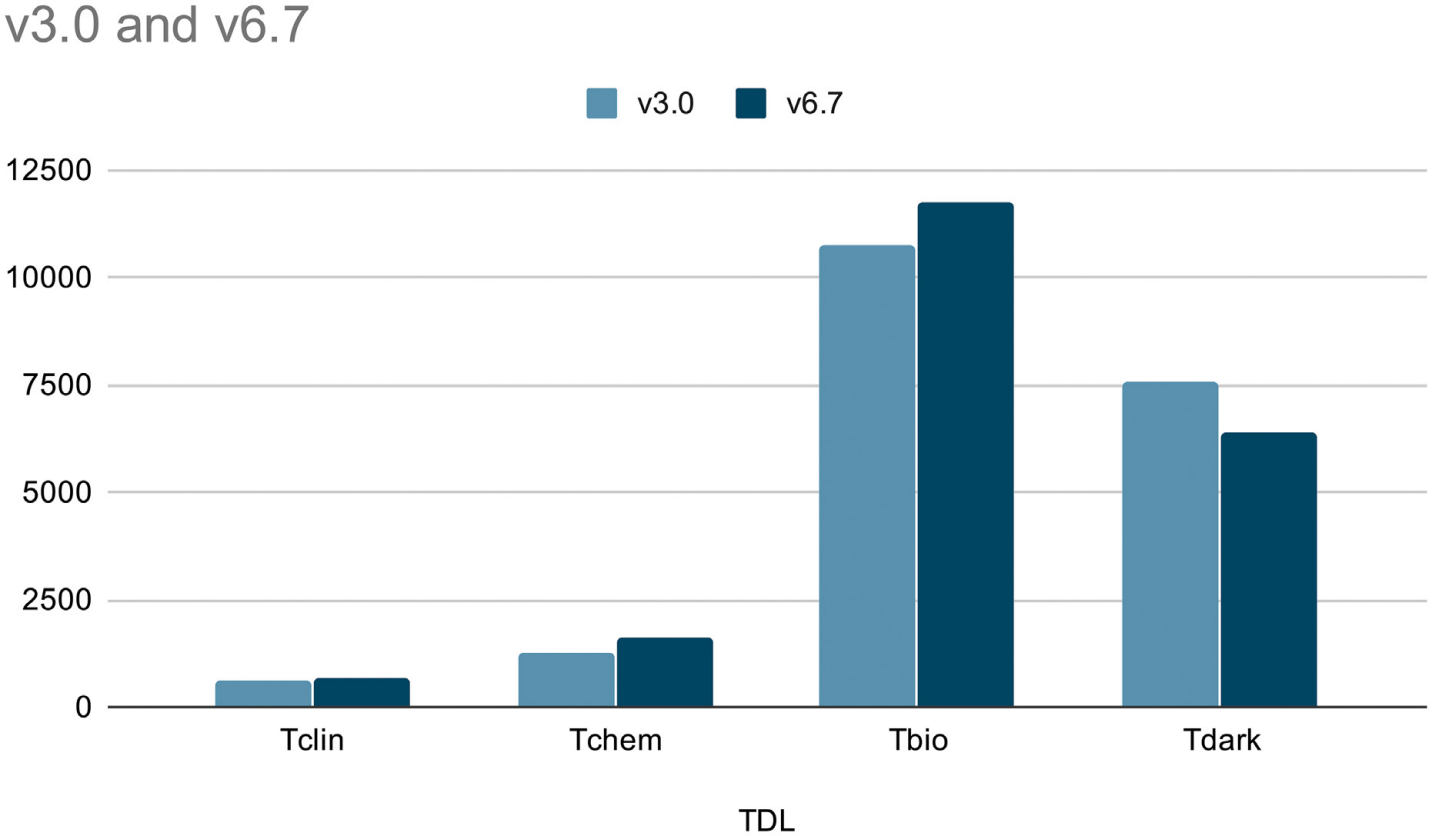
Chart of the TDL changes between TCRD v3.0 and v6.7. The decrease of Tdark and subsequent increase of other development levels shows an overall increase in target illumination.

In addition to the new data from the aforementioned sources, TCRD has been continuously updated when newer versions of source databases (e.g. ChEMBL (21), DrugCentral (22), JensenLab PubMed scores (23) are released. As the amount of data available for each target changed with each release of TCRD, the target development levels (TDLs) for respective targets were recalculated when the target criteria changed. This was done using automated scripts. Very briefly, the TDL is one of four potential values: Tclin, Tchem, Tbio or Tdark. **Tclin** are protein drug targets via which approved drugs act (24–26), which currently includes 659 human proteins; **Tchem** are proteins that are not **Tclin**, but are known to bind small molecules with high potency (currently *N* = 1607); **Tbio** includes proteins that have Gene Ontology (27) ‘leaf’ (lowest level) term annotations based on experimental evidence; or meet two of the following three conditions: A fractional publication count (28) above 5, three or more Gene RIF, ‘Reference Into Function’ annotations (https://www.ncbi.nlm.nih.gov/gene/about-generif), or 50 or more commercial antibodies, as counted in the Antibodypedia portal (29). The fourth category, **Tdark**, currently includes ∼31% of the human proteins that were manually curated at the primary sequence level in UniProt, but do not meet any of the **Tclin**, **Tchem** or **Tbio** criteria. Figure 1 shows the TDL count changes between version 3 and 6.7.

For a further in depth exploration of each additional TCRD dataset and its database changes, we direct the reader to Supplementary Materials.

## RESULTS

In the first 2 years following the initial release, the Pharos team frequently demoed the site, with a focus on obtaining user feedback, which provided the user-centered design information needed as Pharos underwent a ground-up rewrite in late 2018. By focusing on user needs, such as improving the target details page hierarchy and navigation, and adding more detailed explanations of the terms and ideas represented within Pharos, we were able to streamline target presentation, and simplify many pages. Many tables were swapped with sortable list elements, akin to a shopping site, allowing more data to be shown than a table would allow and also allowing us to display dynamic data as desired. Pharos was also redesigned with a focus on mobile usability, adjusting styles and visualizations depending on the screen used. Pharos is also installable as a Progressive Web App, which allows users to install Pharos as a mobile application on their device, increasing the ease of access for Pharos.

To increase speed and responsiveness, we switched from a fully server-side rendered application to a hybrid client and server-side application, allowing faster page rendering and data retrieval. We replaced the REST API with a GraphQL instance, which adds flexibility to data retrieval, as well as potentially reducing the amount of data being sent over the network. A discussion of GraphQL benchmarking is summarized in Supplementary Table S1. Documentation for the GraphQL format, as well as an interactive sandbox with several sample queries can be found at https://pharos.nih.gov/api.

### Browse pages

**Figure 2.**
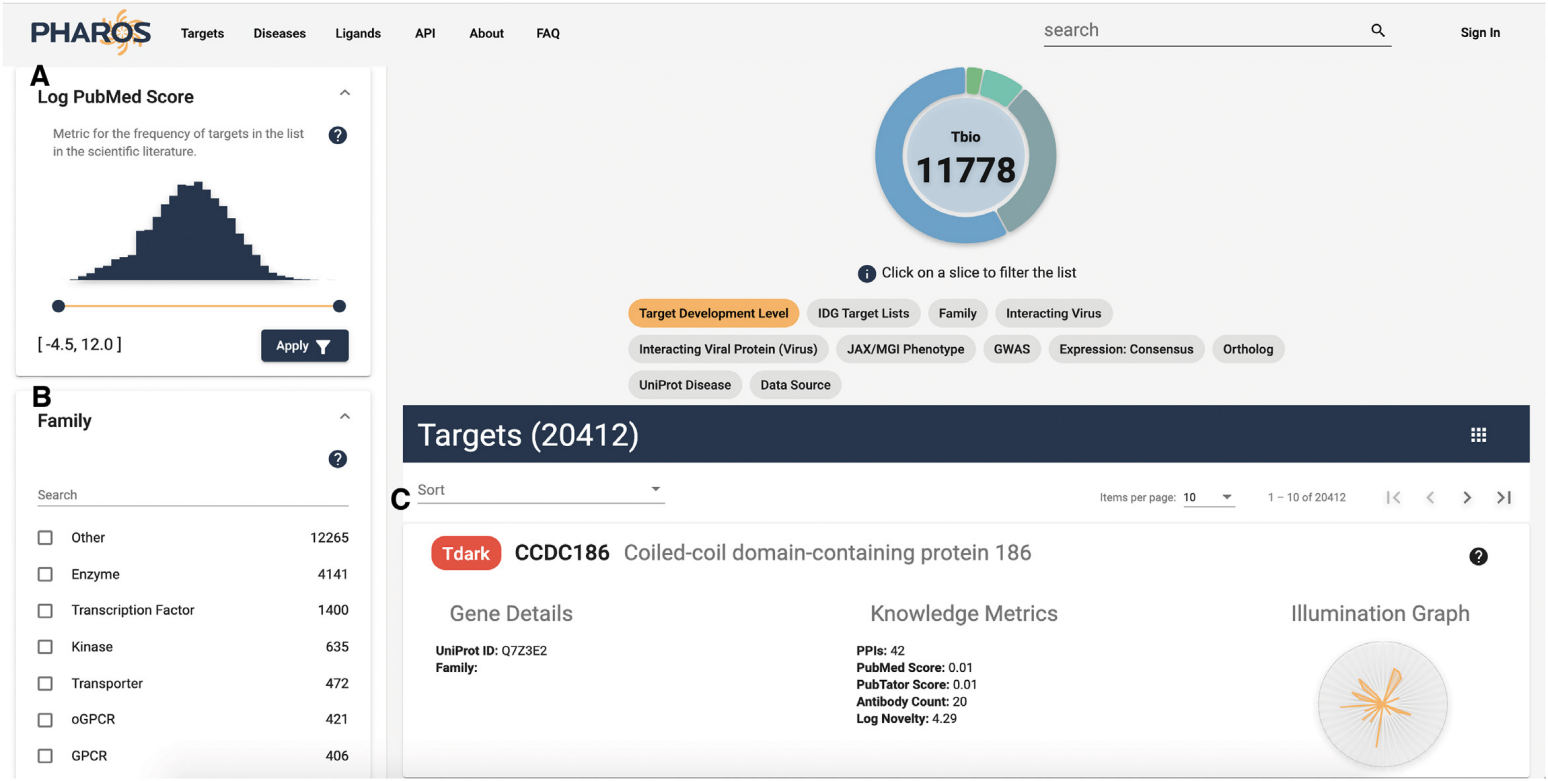
Browse targets page, showing new numeric slider facets for Log PubMed Score range (**A**), searchable protein Family filter panel (**B**) and improved target card view (**C**). The Log PubMed Score filter also shows the definition section displayed.

Filter functionality has been expanded, and users are able to examine the entire list of values for each filter, as well as search for text within the filter values. In the case of numeric values, such as novelty and PubMed score, a range slider allows users to refine results. All filters also have a ‘**?**’ help button that allows users to view a quick definition of the filter or to visit the original source, if desired. These features are displayed in Figure 2. In addition, sub-lists are generated on entity (target/disease/ligand) pages that can be viewed in their respective list browsers. It is therefore possible to browse the list of disease or ligands associated with a specific target, or targets associated with a disease or ligand. For associated diseases, additional numeric filters such as association score and interaction scores are available, as well as ligand affinity measurements for associated ligand lists.

By registering, users are able to select targets of interest and save them as custom lists, which are added to the main filter panel, and are always available. This allows users to further refine a filtered list of targets, as well as view how this new list may be further filtered, or examine the makeup of the list by the various filter categories.

### Target details pages

**Figure 3.**
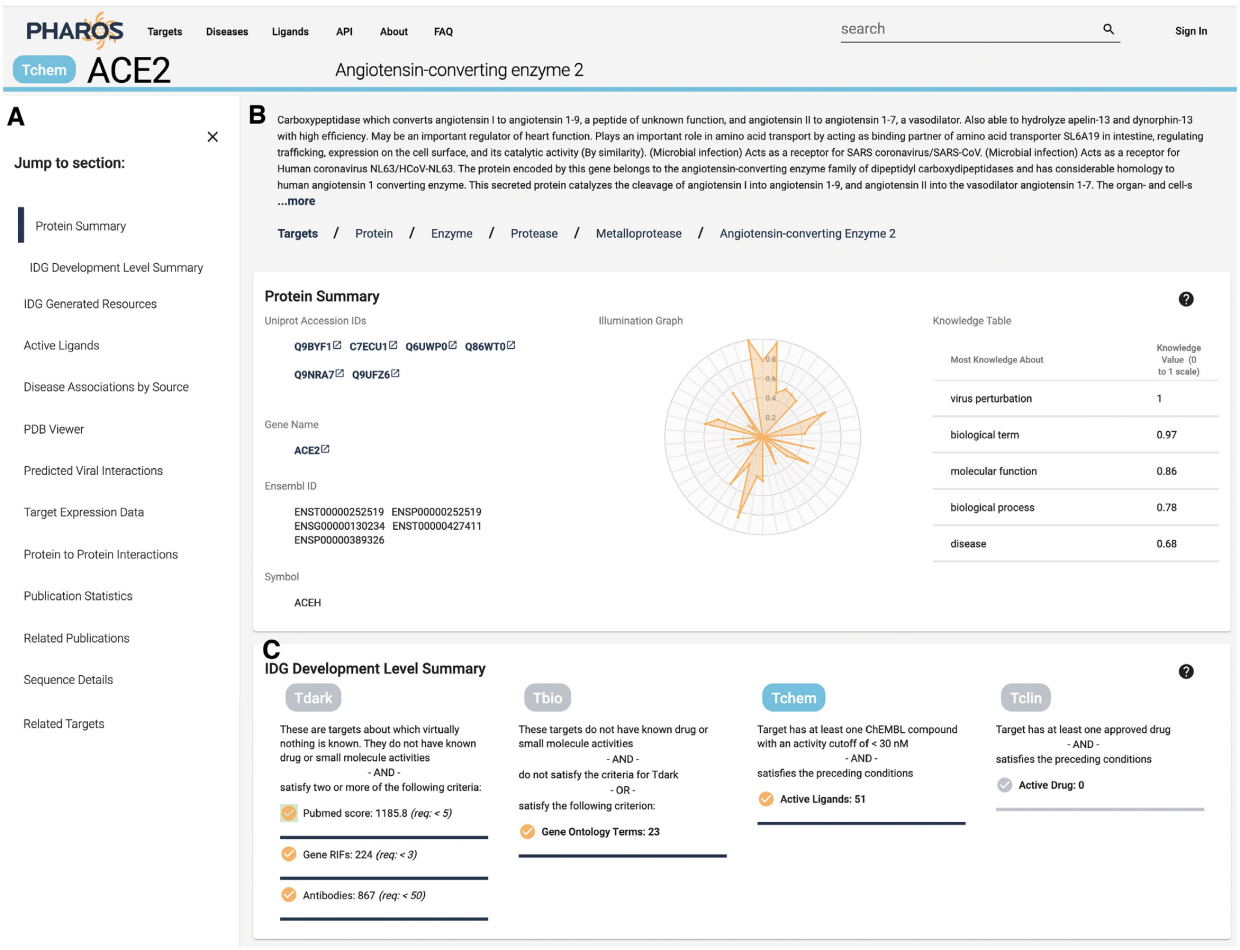
Target details view for ACE2, showing the new table of contents section (**A**), Target overview section (**B**) and TDL section (**C**).

In improving the target details pages, we relied on user interviews that we had conducted to determine the optimal layout and ordering of data on the page. In the newest version of Pharos, target detail pages start off with synonyms, identifiers and a broad overview of the knowledge about the target, as shown in Figure 3. Next, the user is able to examine the TDL criteria in relation to the specific target. Drug and ligand data comes next, followed by disease association data. Target expression and interaction data follows, with publication data, amino acid sequence information – including the ProtVista (30) graphical representation, and filters for related targets completing the page. Sections where no data are available, such as drug and ligand browsers for **Tdark** and **Tbio** targets, are automatically removed. The navigation panel is always visible on the left hand side, allowing users to easily jump to the section of interest. Most data panels have been improved by adding tooltips to further explain properties, and a help panel that contains additional information, such as definitions, explanatory articles or raw data. Additional panels have been added to display additional data.

#### TDL descriptions

One addition to the previous Pharos UI is a description panel. While the various TDL criteria are well documented, it was not apparent to users how, and which TDL criteria are met by a given target. This section displays which criteria have been met, as well as the level or score. A highlighted checkbox indicates which TDL criteria have been met. This provides valuable evidence that illustrates to the user how TCRD generated this ranking. For **Tdark** and **Tbio**, this is also a useful indicator of what criteria are still deficient, and how close to the cutoff they are, potentially driving research in those areas. This panel is also featured in Figure 3.

#### IDG resources

**Figure 4.**
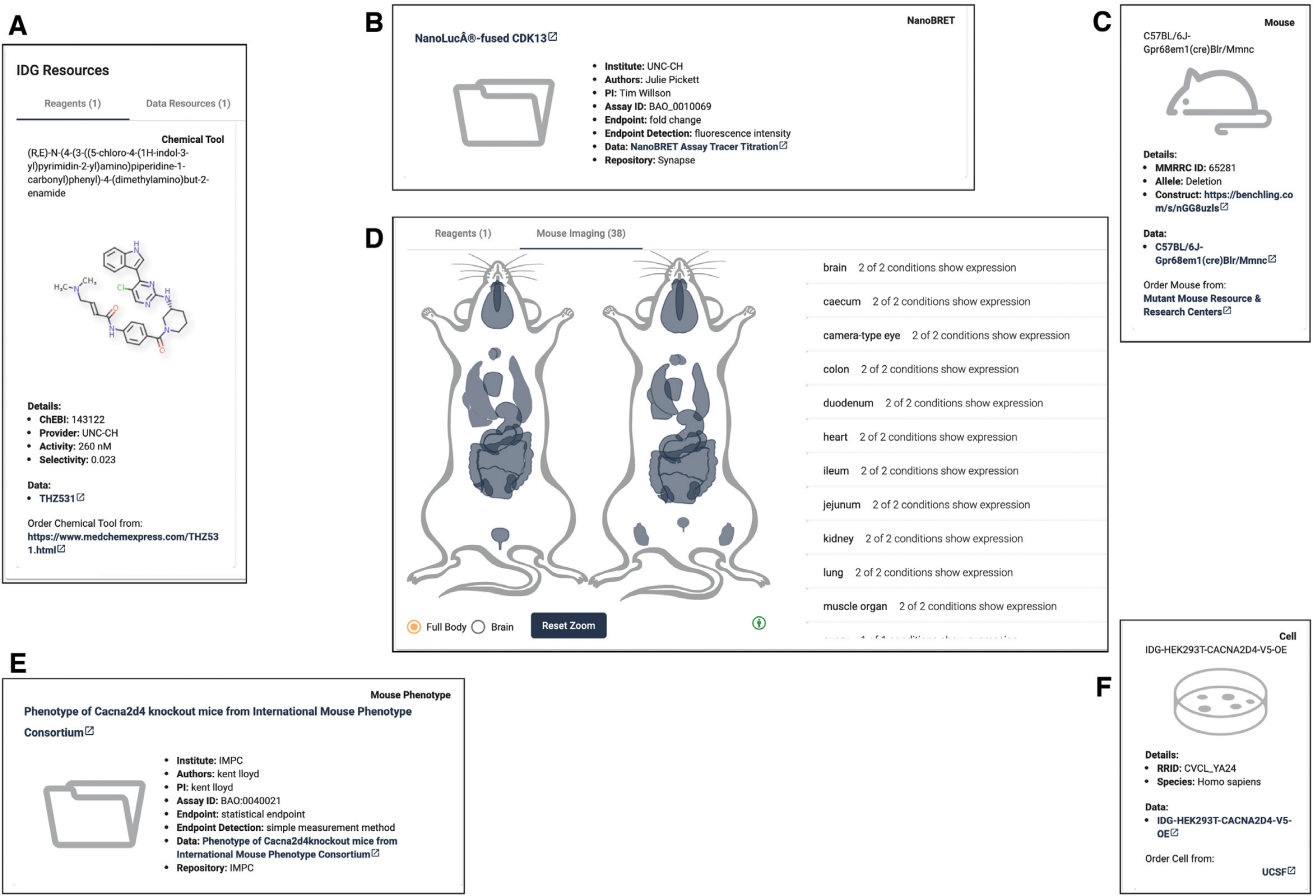
IDG generated resources for ACE2, consisting of small molecule reagents (**A**), and data (**B**). Targets with mouse cell lines, such as GPR68, also have information about that resource (**C**) as well as a mouse tissue expression viewer (**D**). Other data can include, such as the case for CACNA2D4, mouse phenotype (**E**) and cell line (**F**) data. Supplementary Table S2 contains a full breakdown of data types and fields collected.

A feature of the IDG program that has expanded since the initial publication is that of new data or reagents, which are generated by IDG Consortium members. Where data or reagents are available, a browseable section is displayed in Pharos, allowing users to navigate to the data set, and even order them in the case of physical resources, as shown in Figure 4. For targets with mouse expression data, we have incorporated our anatamogram image (discussed below) using male/female and brain mouse images. We refer the reader to Supplementary Materials for an in depth discussion of IDG Consortium resources available in Pharos.

#### Improved ligand/drug section

Drug and ligand browsing is now done via a pageable list. Users can now page through the entire list of approved drugs or active ligands, or open the entire list in the browse view, allowing for filtering of these lists, while specifying the number of targets each ligand is active on.

#### Disease associations

The disease associations panel has been greatly expanded, both in the amount of featured resources, now incorporating DisGeNet (31) and eRAM (32), as well as in size and placement. The source of each disease association is displayed as a collapsible panel that bundles multiple sources for the same disease. Upon expanding this panel, users are able to examine the evidence used to make this association. This allows users to discover provenance, an essential attribute of data aggregated in TCRD. It is also possible to view the list of associated diseases as a browseable list, again, allowing for filtering and more refined data analysis.

#### PDB visualizations

**Figure 5.**
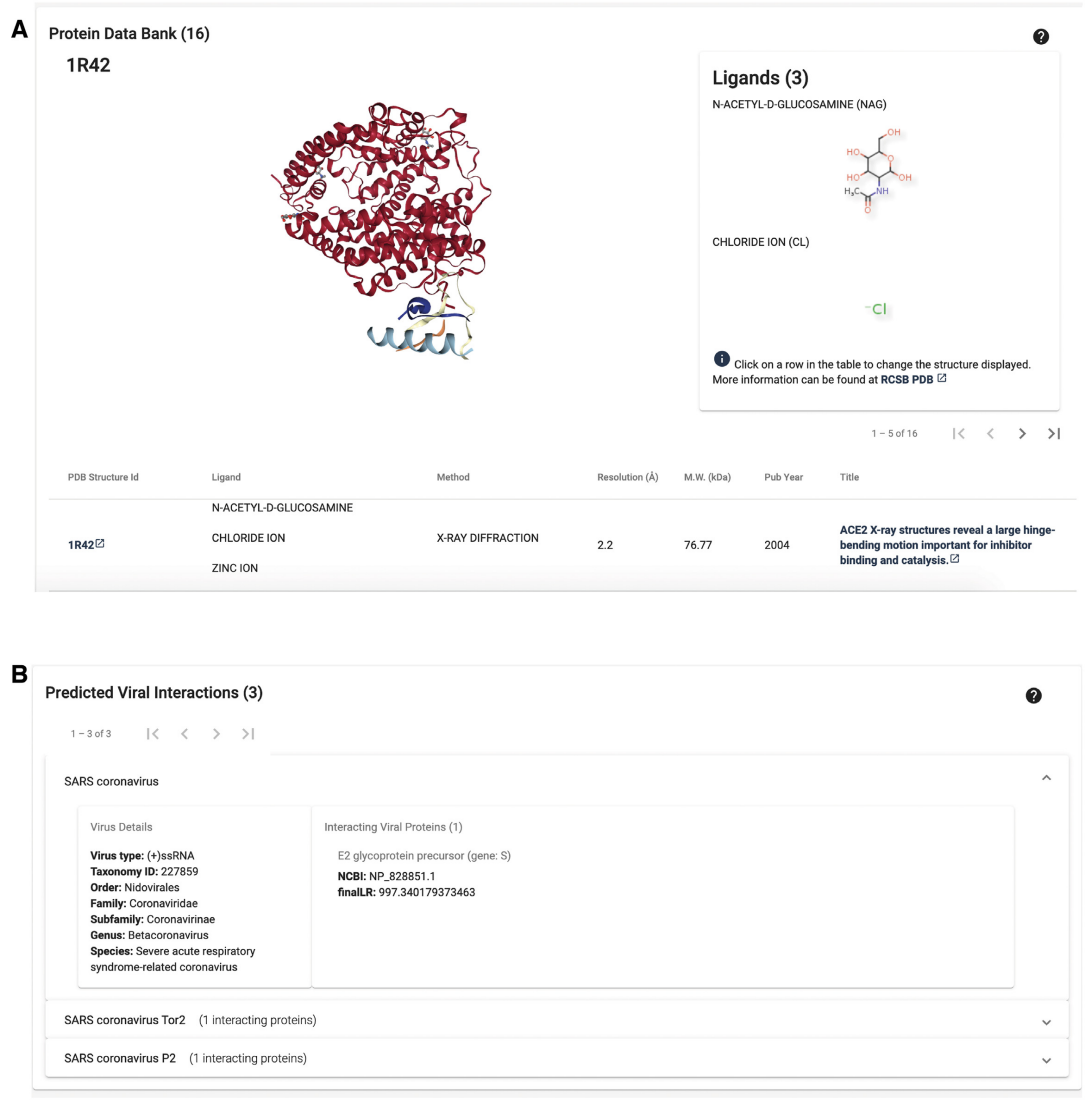
Target details view for ACE2, showing the improved Protein Data Bank data viewer (**A**) and predicted viral interactions (**B**).

While PDB identifiers have always been available in Pharos, they were previously displayed as a list of linkouts with no additional information (33). In Pharos 3.0, the PDB section was expanded and redesigned to display three-dimensional (3D) structures for proteins, including bound ligands where available. This 3D visualization, employing NGL Viewer (34,35), is highly interactive, allowing users to drag, zoom, pan and spin the structure to thoroughly examine it. This interface also enables users to browse the PDB identifiers and structures associated with a given target. Clicking on a table row loads the respective PDB entry, and displays the ligands shown in the structure. Other details include the reference source for the id, as well as the method used to generate the structure. This feature is highlighted in Figure 5.

#### Predicted viral interactions

Predicted viral-human protein interactions from the P-HIPSTer atlas are displayed in a separate panel that shows a list of viruses and their associated taxonomic data, specifically for the targeted human protein. Each virus also lists the viral proteins that the human protein is predicted to interact with, in addition to a likelihood ratio, which measures the strength of the sequence- and structure-based prediction. These features are shown in Figure 5, using ACE2, the angiotensin converting enzyme 2, as example. ACE2 is the functional entry receptor for both severe acute respiratory syndrome (SARS) coronaviruses, SARS-CoV (36) and SARS-CoV-2 (37).

#### Target expression data

**Figure 6.**
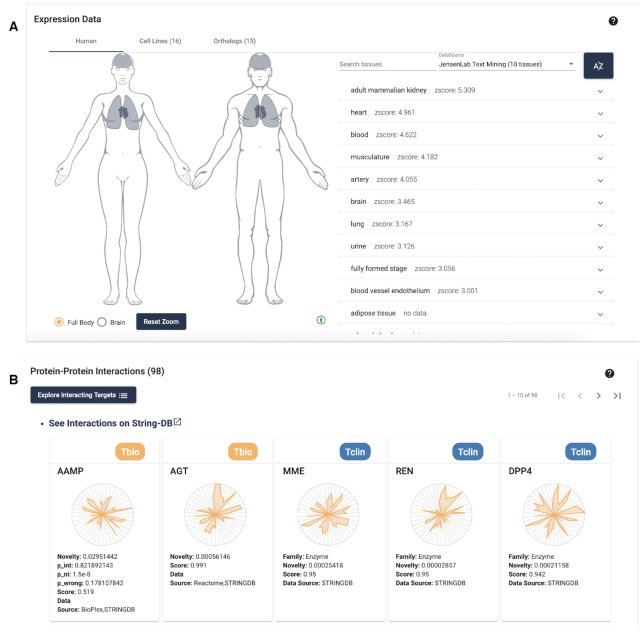
Target details view for ACE2, showing the tissue expression section, with highlighted tissues (**A**), and protein to protein interaction section (**B**). The frequency of updating for resources integrated in TCRD differs. Text-mined sources are updated more frequently, and changes in the scientific literature allow us to track certain associations sooner. Therefore, selecting one of these sources (as displayed) will show that ACE2 is expressed in the lungs.

The target expression data panel has also been expanded, as shown in Figure 6. We replaced the static human figure with an expanded, interactive anatomogram (18). This visualization contains both male and female figures, allowing sex-specific tissue information (e.g., from GTEx) to be highlighted. Users can toggle the brain image separately, for more fine-grained brain tissue expression visualization. All figures are zoomable and pannable, to improve visibility. When possible, all source tissues have been mapped to standardized Uberon (38) identifiers, enabling us to merge repetitive tissues. Expression data can be viewed by source, and tissue values are searchable. Clicking on a tissue panel, like the disease associations section, allows users to view detailed provenance of the data, both showing the source and relevant confidence values for the tissue expression. Gradient coloration allows us to display the tissue expression level, either a qualitative value (low medium, high), or a numeric value scale, depending on the data source, giving users a quick visualization of this data. We note that several sources have an overlap in data and content; however, they may also present unique data. Pharos displays the variety between sources, which can help users decide what sources to focus on. It also illustrates that the text-mining-based resources tend to be updated more often than frequently accessed scientific databases that aggregate experimental data. When available, cell lines and orthologs are displayed with expandable evidence panels.

#### Protein–protein interactions

The introduction of data from Bioplex (39), STRING and Reactome (40) has allowed us to display rich PPI data, and allows users to examine how multiple targets may interact, as shown in Figure 6. This panel consists of a pageable list of targets known to interact with the current target. These targets display the illumination graph, which gives users a quick visual representation of the level and types of knowledge available for the target. The originating data source is also displayed, as well as the confidence of this interaction. This list of PPIs may be viewed either directly in STRING, or viewed in the browse page as a filterable list.

### Disease details pages

**Figure 7.**
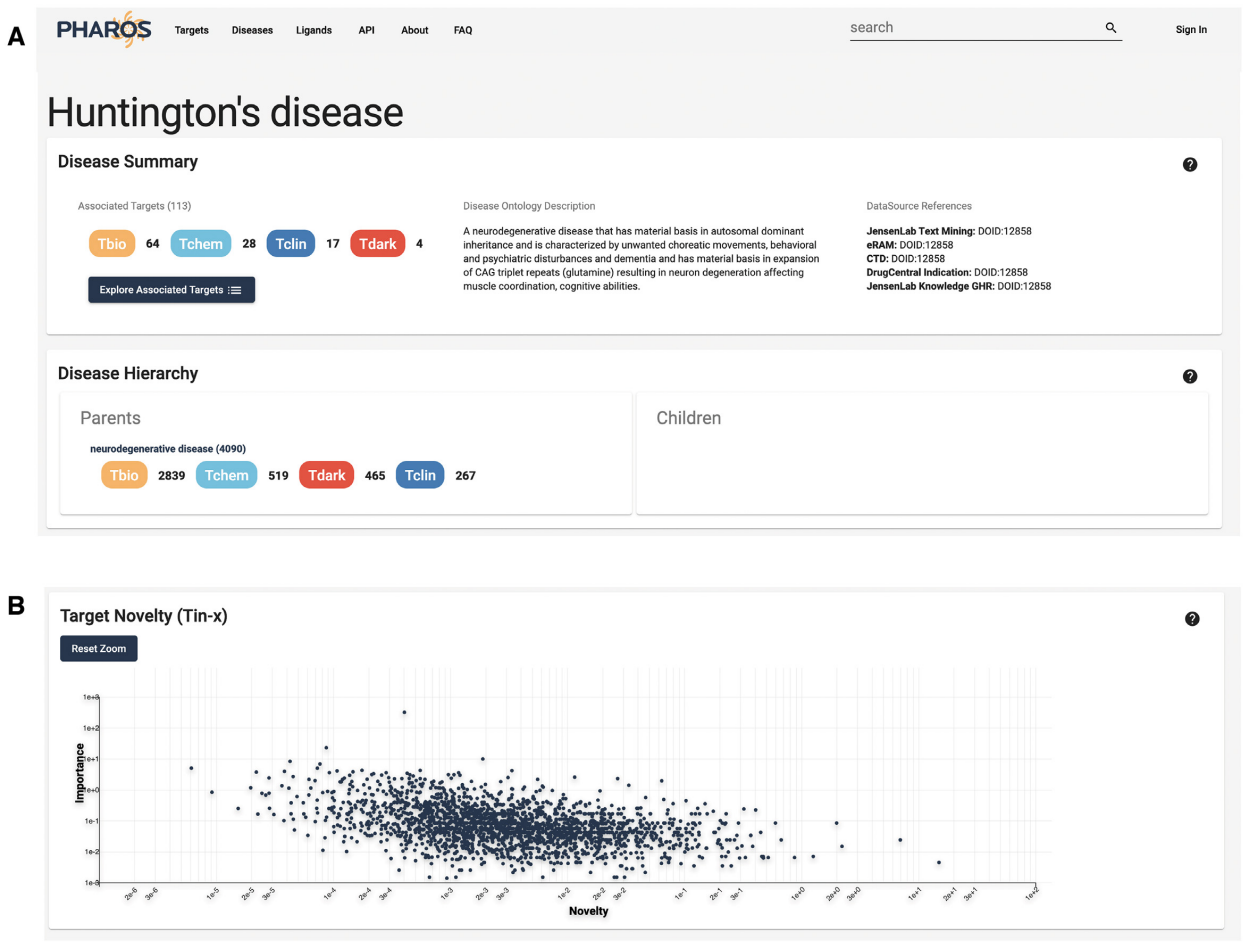
Disease details page for Huntington's disease, showing Disease Ontology description and hierarchy (**A**), and TIN-X plot showing novelty targets and their importance, mapped with a log scale (**B**).

While Pharos remains target-centric, it is equally important to be able to examine diseases in relation to their associated targets. As such, Pharos has always included diseases as a browseable list, as well as a disease details page. Pharos 3.0 expands on the data collected and displayed about diseases. When available, a description from the Disease Ontology is shown. The hierarchy of each specific disease is also shown, allowing users to view parent or child diseases or syndromes, as well as the siblings for each disease. In addition, a breakdown of the targets associated with each disease is shown, which can also be opened in the target browse page, allowing users to filter the list of associated targets. The target-disease Novelty score is shown in the Target-Importance Novelty eXplorer (TIN-X, (41) panel. Similar to the target based TIN-X view, this is a scatterplot of novel targets related to the disease of interest. *Novelty* estimates the scarcity of publications about a target, whereas *Importance* estimates the strength of the association between that target and a specific disease (42). The *X*-axis shows the Novelty of the target-disease relationship, while the *Y*-axis shows the Importance of that target to the disease, both on a log_10_ scale. This feature is displayed in Figure 7. When available, help definition buttons are also shown for each section of data.

### Ligand details pages

**Figure 8.**
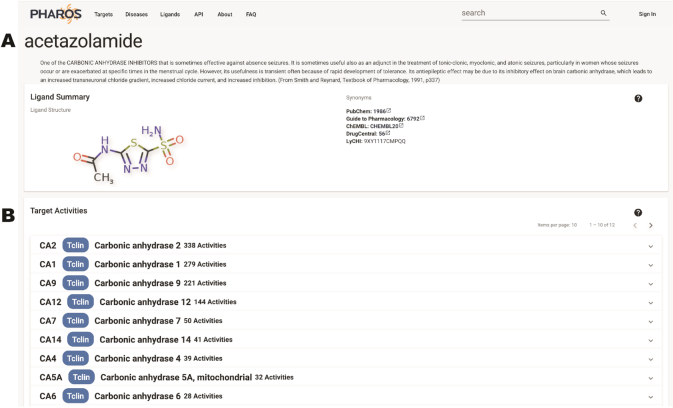
Ligand details page for acetazolamide, shows ligand description information as well as synonyms and identifiers (**A**). Target activity is shown with expanded activity for CA2 (**B**).

The ligand details page has been expanded to include a description of the ligand, where available. A 2D depiction of the ligand chemical structure is shown, together with synonyms and identifiers from PubChem (43), Guide to Pharmacology (44), ChEMBL and DrugCentral. Activity values are displayed in collapsible panels, and sorted by target. By clicking on a target header, users are able to examine activity values for that ligand on the selected target. Activity type, value and mechanism of action are shown, in addition to reference publication for each activity, allowing users to directly view the source. Figure 8 illustrates this page display.

### Use cases

#### Target list search

**Figure 9.**
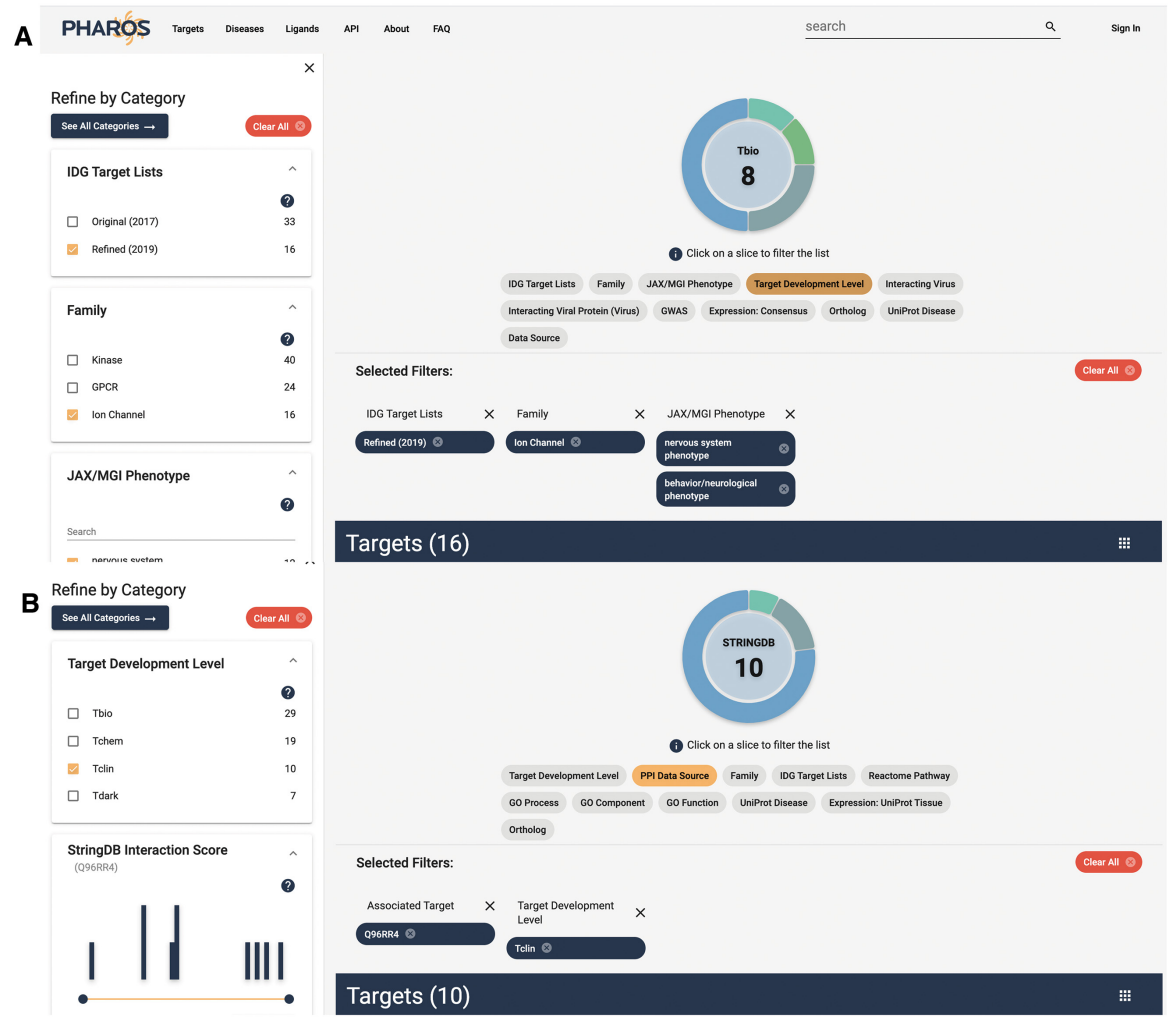
Target list described in *Target List Search* use case (**A**), and target list described in *Binding partner search* use case (**B**). Both panels show selected filters, including the ability to filter target lists by IDG featured sub-lists.

A neuroscientist who studies ion channels is seeking understudied proteins for a student project. Filtering to targets listed by the IDG Consortium as targets of interest (https://druggablegenome.net/IDGProteinList), in the ion channel family, with a nervous system phenotype as described by the Mouse Phenotype Ontology from the Jackson Laboratory yields 16 potential targets of interest. The refined list view is shown in Figure 9.

#### Binding partner search

A scientist is studying the function of CAMKK2, and hypothesizing on its role in behavior. From the CAMKK2 target details page, she clicks ‘Explore Interacting Targets’ to open the potential binding partners in the Target List. Selecting **Tclin**, she filters the list to 10 potential binding partners that have approved drugs, with new therapeutic effects and drug-related side effects that can assist in her research. She is also able to save this list of targets as a custom list, allowing her to easily revisit and analyze these targets. This workflow is also shown in Figure 9.

#### Disease-based search

**Figure 10.**
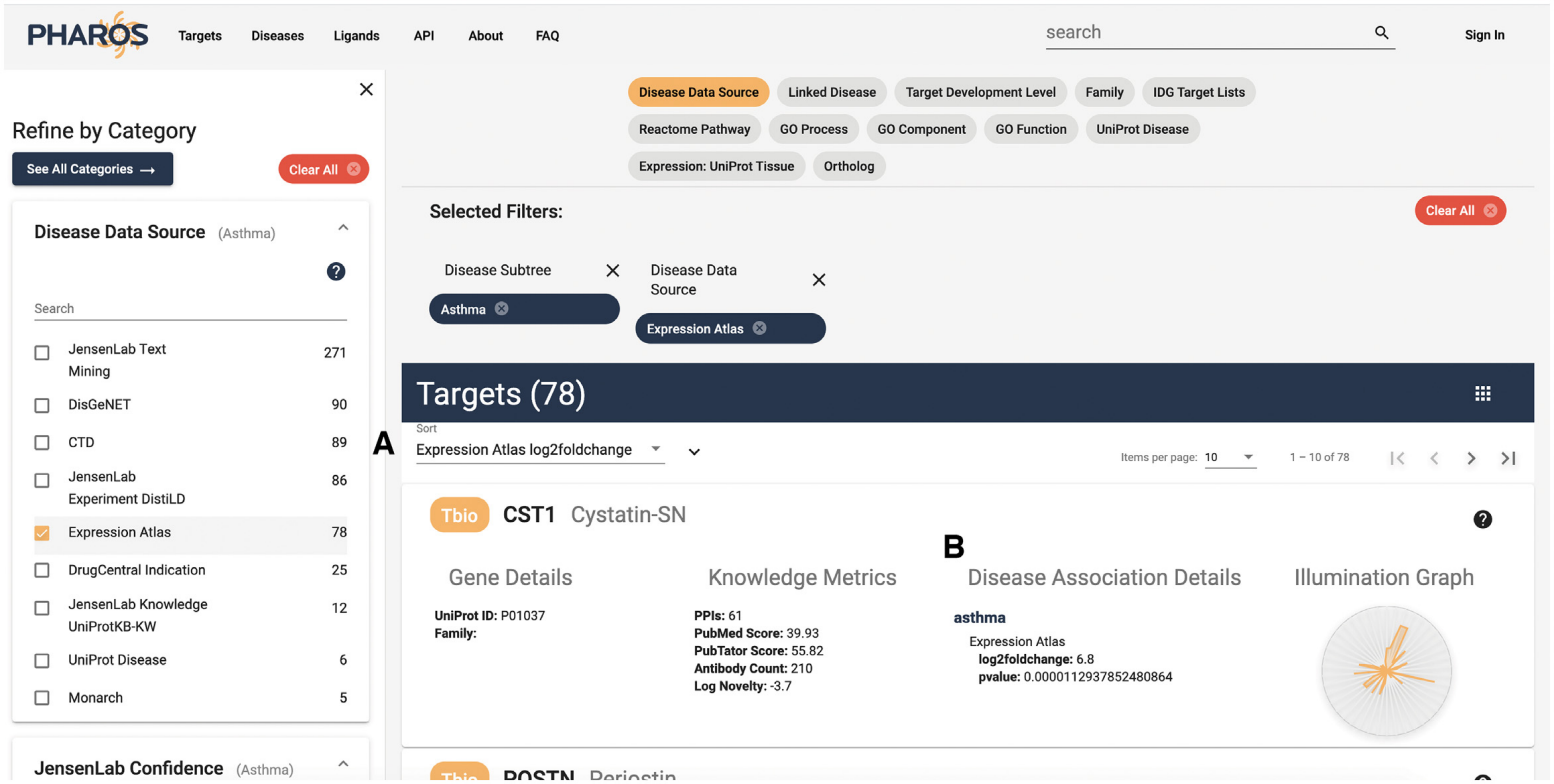
List of targets associated with asthma, filtered by Data Source: Expression Atlas, and filtered by Expression Atlas log2foldchange value (**A**). Also shown is the Disease Association Details column, with additional association-specific details (**B**).

A researcher studying asthma searches for this top level disease term and enters the asthma disease details page. He wants to find proteins that are differentially expressed in asthma patients. Starting from the Asthma disease details page, he clicks ‘Explore Associated Targets’ to see 470 potential targets. Filtering those with data from the Expression Atlas (18), using the ‘Expression Atlas’ value from the ‘Disease Data Source’ filter, yields 78 targets, which can then be sorted by log_2_foldChange using the drop-down sort feature, as shown above the target list in Figure 10. He finds several with high log_2_foldChange, meaning the expression level changes drastically between disease and non-disease conditions, and low p-value, meaning the results are statistically significant. Also of note, for lists of associated targets, an additional column has been added to the target card, showing disease association details, such as the evidence associated with a given data source.

#### Finding experimental tools

A researcher has found that a Voltage-gated potassium channel, KCNS2, is expressed in the nucleus they are working on. It's role is unknown. The target details page for KCNS2 lists three approved drugs (e.g. amifampridine) which are known to block this channel. The IDG Resources show a cell line and a mouse genetic construct, which can be ordered from the IDG Consortium (UCSF, in this case). The researcher is now able to design a set of *in vitro* and *in vivo* experiments to help elucidate the role of the channel.

#### Machine learning based on PPI networks to prioritize targets for a disease

A novel target ranking approach could be developed that relies on deep network representation learning of a PPI network annotated with disease-specific knowledge attributes derived from TCRD/Pharos. This involves mapping the enriched PPI network into a feature space using the neural network framework, Gat2Vec (45). Gat2Vec employs a shallow neural network model to facilitate joint learning on the structural and attribute contexts of a given network. In this case, the PPIs provide the structural context and the disease-related knowledge provides the attribute contexts. The feature space generated can be used to develop machine learning models that can predict the ranking of protein targets in the context of a disease. Additional protocols for extracting available data for specific targets of interest, as well the differences in knowledge availability between understudied and more studied targets, are discussed elsewhere (46).

## DISCUSSION

With the newly added data, interactive visualizations and enhanced search capabilities, TCRD and Pharos in their current forms serve as IDG resources that facilitate better exploration of the dark and understudied regions of the human genome. The central idea of the resource continues to be enriching knowledge around human targets and monitoring their therapeutic development levels. One area that received significant attention in the current update is the search mechanism which was upgraded with the implementation of GraphQL API. To improve user interaction with the new API, example queries have been made available on the website. The TDL descriptions panel and the target expression anatomograms are the significantly improved components of the Pharos web interface. Further, most data that have been recently integrated into TCRD facilitate application of artificial intelligence/machine learning (AI/ML) techniques for target evaluation and drug repositioning (47), and are accessible in ML ready format. Most of the above described developments are based on the feedback obtained directly from the website visitors, several demo sessions and presentations at scientific meetings.

Ongoing work focuses on utilizing the AI/ML-ready data such as target-disease, target-phenotype and PPI data to develop AI/ML models for prioritization of dark targets and better understanding of disease biology. Currently, we are evaluating ways to aggregate experimental data uncertainties, specifically data quality and reliability (48), similar to the *in silico* toxicology protocols (49). We continue to extend our efforts in enhancing the disease page to list associated targets, rank them by the consensus strength of association to a disease and further facilitate filtering to narrow down to targets of potential interest to researchers. The database can be expected to be further enriched with new data and data types that contribute to the ultimate goal of illuminating the dark targets.

## DATA AVAILABILITY

TCRD is an open source database that can be accessed at

http://juniper.health.unm.edu/tcrd/

Pharos is an open source web platform that can be accessed at

https://pharos.nih.gov/

The Pharos resources have been split into frontend and backend repositories.

The front end code can be found on Github.

https://github.com/ncats/pharos_frontend

The backend GraphQL implementation code can be found on Github.

https://github.com/ncats/pharos-graphql-server

GraphQL resource documentation can be found on Pharos

https://pharos.nih.gov/api

## Supplementary Material

gkaa993_Supplemental_File
