# Supplementary material for: TCRD and Pharos 2021: mining the human proteome for disease biology
Source: Nucleic Acids Res. 2020 Nov 6;49(D1):D1334–46. doi: 10.1093/nar/gkaa993 (PMC7778974; doi:10.1093/nar/gkaa993)
Supplement: gkaa993_Supplemental_File [file gkaa993_supplemental_file.docx]

*Supplementary Material for*

**TCRD and Pharos 2020: Mining the Human Proteome for Disease Biology**

Timothy K. Sheils^1^, Stephen L. Mathias^2^ , Keith J. Kelleher^1^, Vishal B. Siramshetty^1^, Dac-Trung Nguyen^1^, Cristian G. Bologa^2^, Lars Juhl Jensen^3^, Dušica Vidović^4,5^, Amar Koleti^4^, Stephan C. Schürer^4,5,6^, Anna Waller^7^, Jeremy J. Yang^2^, Jayme Holmes^2^, Giovanni Bocci^2^, Noel Southall^1^, Poorva Dharkar^1^, Ewy Mathé^1^, Anton Simeonov^1^, and Tudor I. Oprea^2,3,8,9,*^

^1^ National Center for Advancing Translational Science, 9800 Medical Center Drive, Rockville, MD 20850, USA

^2^ Translational Informatics Division, Department of Internal Medicine, University of New Mexico Health Sciences Center, Albuquerque, NM 87131, USA

^3^ Novo Nordisk Foundation Center for Protein Research, Faculty of Health and Medical Sciences, University of Copenhagen, 2200 Copenhagen, Denmark

^4^ Institute for Data Science and Computing, University of Miami, Coral Gables, FL 33146, USA

^5^ Department of Molecular and Cellular Pharmacology, Miller School of Medicine, University of Miami, Miami, FL 33136, USA

^6^Sylvester Comprehensive Cancer Center, Miller School of Medicine, University of Miami, Miami, FL 33136, USA

^7^ UNM Center for Molecular Discovery, University of New Mexico Health Sciences Center, Albuquerque, NM 87131, USA

^8^ UNM Comprehensive Cancer Center, University of New Mexico Health Sciences Center, Albuquerque, NM 87131, USA

^9^ Department of Rheumatology and Inflammation Research, Institute of Medicine, Sahlgrenska Academy at University of Gothenburg, Gothenburg, Sweden

* To whom correspondence should be addressed. Tel: +1 505 925 7529; Fax: +1 505 925 7625; Email: toprea@salud.unm.edu

**Description of TCRD Updates, V3 to V6**

**1. TCRD v3 to v4.**

*Provenance:* A critical change in TCRDv4 was storing provenance for all TCRD data. The granularity of data provenance depends on the incorporated dataset, so TCRD stores data provenance at the table, column and/or row level as appropriate for each dataset. TCRD includes 128 descriptions of provenance associated with its 77 different datasets.

*LocSigDB:* A comprehensive compendium of manually curated targeting signals for eight distinct subcellular locations. TCRD contains 106521 location, signal, and associated PubMed ID information from LocSigDB that map to 18916 targets in TCRD.

*Orthologs:* TCRD includes HGNC orthologs from 16 species: Chimp, Macaque, Mouse, Rat, Dog, Horse, Cow, Pig, Opossum, Platypus, Chicken, Anole lizard, Xenopus, Zebrafish, C. elegans, Fruitfly and S. cerevisiae. These are stored in TCRD if at least 2 out of 3 of the sources Inparanoid, OMA, and EggNOG agree on this ortholog assignment. Currently there are 178,759 orthologs associated with 18,056 distinct TCRD targets.

*GeneRIF Years:* TCRD now stores year(s) corresponding to PubMed ID(s) associated with each GeneRIF. TCRD contains a total of 740,376 GeneRIFs associated with 16,411 targets, of which 721650 have year(s).

**2. TCRD v4 to v5.**

*GuideToPharmacology.* TCRD stores small molecule bioactivities from GuideToPharmacology in addition to ChEMBL activities. TCRD now includes 11,191 activities from GuideToPharmacology that are associated with 1,321 unique proteins.

*eRAM.* TCRD now contains disease associations from the eRAM encyclopedia for rare diseases. TCRD contains 14,643 new disease associations from eRAM for 5,686 unique proteins.

*Uberon.* TCRD now contains the Uberon ontology and we have attempted to map all expression data to an Uberon ID (with limited success). Currently 3,365,748 of 26,935,759 (12.5%) expression rows in TCRD have an Uberon ID.

*DRGC Resources.* TCRD includes data describing resources produced by the IDG DRGCs. Currently, TCRD has 18 resources of type Small Molecule associated with 18 distinct targets.

**3. TCRD v5 to v6.**

*Mouse and Rat Proteins.* Mouse and Rat proteins from UniProt have been added to the new table nhprotein. TCRD has 85,187 mouse proteins and 36,090 rat proteins.

*Mouse Phenotype Associations.* TCRD contains 7,245,935 gene-phenotype associations from IMPC, which are associated with 27,637 unique mouse genes.

*Rat QTL Associations.* TCRD contains 1799 QTLs from RGD associated with 1,799 unique rat genes.

*Ontologies.*

- TCRD previously included Disease Ontology data; it now includes 36,392 cross-references to 26 distinct external disease ontologies/terminologies.
- TCRD now contains Rat Disease Ontology data, specifically 18,085 terms and 39,167 cross-references to 26 distinct external disease ontologies/terminologies.
- TCRD now contains 12,894 Mammalian Phenotype Ontology terms.
- Previously, TCRD included Uberon Ontology data; it now also includes 35,043 cross-references to 69 distinct external ontologies/terminologies.

*GWAS Catalog.* TCRD incorporates a new GWAS table (no longer in the phenotype table). This table incorporates EBI GWAS Catalog data; this has been expanded to include additional fields.

*GTEx.* GTEx expression data has been expanded to include sex-specific values and sex-specific tissue specificity scores. This now resides in the new table gtex, no longer in the expression table.

*OMIM.* Added new tables omim and omin_ps for MIM phenotypes and phenotype series IDs and titles, respectively.

*STRING-db*: TCRD now includes 11,638,404 protein-protein interactions - including scores - from STRING 11.

*Homologene* data for human, mouse and rat has been added to TCRD, specifically 69,991 orthologs associated with 18,806 unique targets. TCRDv6 now has homology data from Homologene in addition to our in-house generated homology data in the ortholog table.

*CCLE* TCRD now includes expression data from the Cancer Cell Line Encyclopedia, specifically 21,716,003 values associated with 18,750 unique human proteins.

*ClinVar* TCRD now includes 511,408 phenotype associations from ClinVar associated with 2,947 unique targets.

*LINCS* TCRD now includes cell perturbation expression data from LINCS. These data include 84,097,720 values associated with 980 distinct TCRD targets.

*P-Hipster* TCRD now contains viral protein and human-virus protein-protein interaction data received from Gorka Lasso, and originally incorporated into the human-virus interactome atlas (http://phipster.org/. Specifically, TCRD has 282,528 protein-protein interactions between 5,719 unique human proteins and 7,463 unique viral proteins.

*Ongoing Updates*

TCRD is updated on an on-going basis. When source datasets such as ChEMBL, DrugCentral, JensenLab PubMed Scores are updated, the new versions are incorporated into TCRD, replacing the previous versions, and TDLs are recalculated if appropriate.

**Summary Statistics, TCRD V6**

*TDL*

| **TDL** | **Count** |
| --- | --- |
| Tclin | 659 |
| Tchem | 1,607 |
| Tbio | 11,778 |
| Tdark | 6,368 |

*IDG Families/TDLs (all proteins and IDG eligible targets)*

| **IDG Protein Category** | **TDL** | **Count** | **IDG eligible** |
| --- | --- | --- | --- |
| GPCR (non-olfactory) | Tclin | 100 | N/A |
| GPCR (non-olfactory) | Tchem | 142 | 29 |
| GPCR (non-olfactory) | Tbio | 119 | 55 |
| GPCR (non-olfactory) | Tdark | 45 | 33 |
| Ion Channel | Tclin | 126 | 15 |
| Ion Channel | Tchem | 87 | 4 |
| Ion Channel | Tbio | 105 | 26 |
| Ion Channel | Tdark | 26 | 17 |
| Kinase | Tclin | 65 | 1 |
| Kinase | Tchem | 361 | 87 |
| Kinase | Tbio | 182 | 42 |
| Kinase | Tdark | 27 | 20 |

*Disease Associations indexed in TCRD*

| **Source** | **Protein-disease associations count** | **Unique protein**  **count** |
| --- | --- | --- |
| CTD | 35,187 | 7,837 |
| DisGeNET | 82,875 | 9,025 |
| DrugCentral Indication | 12,853 | 945 |
| eRAM | 14,660 | 5,139 |
| Expression Atlas | 15,9846 | 16,784 |
| JensenLab Experiment COSMIC | 17,267 | 11,303 |
| JensenLab Experiment DistiLD | 6,134 | 3,862 |
| JensenLab Knowledge GHR | 3,683 | 2,313 |
| JensenLab Knowledge UniProtKB-KW | 3,427 | 2,411 |
| JensenLab Text Mining | 56,459 | 13,030 |
| Monarch | 9,509 | 3,825 |
| UniProt Disease | 5,651 | 3,766 |

*Phenotype Associations*

| **Source** | **Gene-phenotype associations count** | **Unique gene count** |
| --- | --- | --- |
| IMPC | 7245935 | 27637 |
| GWAS Catalog | 124149 | 13116 |
| JAX/MGI Human Ortholog Phenotype | 58398 | 10204 |
| OMIM | 14147 | 13856 |
| RGD | 1799 | 908 |
| ClinVar | 511408 | 2947 |

*Gene/Protein Expression*

| **Source** | **Values** |
| --- | --- |
| CCLE | 21,716,003 |
| Cell Surface Protein Atlas | 10,104 |
| Consensus | 206,323 |
| GTEx | 1,805,436 |
| HCA RNA | 1,075,480 |
| HPA | 782,711 |
| HPM Gene | 482,640 |
| HPM Protein | 840,420 |
| JensenLab Experiment Cardiac proteome | 20,027 |
| JensenLab Experiment Exon array | 137,835 |
| JensenLab Experiment GNF | 242,915 |
| JensenLab Experiment HPA | 494,017 |
| JensenLab Experiment HPA-RNA | 304,417 |
| JensenLab Experiment HPM | 200,237 |
| JensenLab Experiment RNA-seq | 124,554 |
| JensenLab Experiment UniGene | 97,003 |
| JensenLab Knowledge UniProtKB-RC | 68,777 |
| JensenLab Text Mining | 62,098 |
| UniProt Tissue | 70,198 |

*Pathways*

| **Source** | **Pathway associations count** | **Protein count** |
| --- | --- | --- |
| KEGG | 32,325 | 7,686 |
| PathwayCommons: humancyc | 1,468 | 779 |
| PathwayCommons: inoh | 7,363 | 1,546 |
| PathwayCommons: netpath | 2,967 | 1,488 |
| PathwayCommons: panther | 5,019 | 2,159 |
| PathwayCommons: pid | 8,249 | 2,597 |
| Reactome | 110,872 | 10,781 |
| UniProt | 1,224 | 1,165 |
| WikiPathways | 162,449 | 6,411 |

*Protein-protein Interactions*

| **Source** | **Interactions count** |
| --- | --- |
| BioPlex | 75,519 |
| Reactome | 21,866 |
| STRING db | 11,638,404 |
| P-Hipster | 282,528 |

*Drug/Compound Activities*

| **Source** | **Activities count** | **Target count** |
| --- | --- | --- |
| ChEMBL | 489,802 | 1,791 |
| Guide to Pharmacology | 11,191 | 1,321 |
| DrugCentral | 4,004 | 993 |

**GraphQL**

While REST APIs remain commonplace among web applications, GraphQL usage is rapidly increasing. In the case of Pharos, as the TCRD grew in size with each subsequent release, it was quickly becoming untenable to return the same JSON object we had been previously returning. By switching to a GraphQL backend implementation, we were able to retain the initial TCRD database as-is, rather than re-create and modify it. The below chart illustrates the changes in returned object size from Pharos 1.0 to 3.0. It is important to note that the REST API and GraphQL database do not return the exact same object. The REST API returns a lightweight object based on parameters displayed on the target list page, or a full object consisting of all linked data. In the case of publications or active ligands, this could be a long list, and that data primarily consisted of a link to the full publication or ligand object, requiring a second API call to fetch the data. GraphQL on the other hand, allows developers to break up the response and paginate sub-sections of the data, meaning that rather than return 100 or more active ligands, the query can be modified to return 10 ligands at a time.

In addition to being able to paginate sections of the query, it is also possible to specify which properties are returned. Instead of receiving a link to the ligand data API call, Pharos 3.0 returns the ligand data required for display, eliminating the need for subsequent API calls. By returning the first page of ligand results in a target, we also minimize the amount of data retrieved, and only fetch those results for users who are interested. An additional feature of Pharos 3.0 is the ability to fetch specific properties. The table below shows the data payload if a user simply wanted the target development level of a target. Rather than fetch the entire JSON object from the database, we are able to return just the field required, thus making data retrieval much more flexible within Pharos, as well as for external users who may want to use these services.

Table S1. A comparison of the payload size of 10 targets using the Pharos 1.0 Rest API and the Pharos 3.0 GraphQL instance. The payload size of a Pharos 3.0 query to retrieve just the target development level is also shown, as well as the target development level itself.

| Target | REST API (kb) | GraphQL (kb) | TDL only | TDL |
| --- | --- | --- | --- | --- |
| ACE2 | 44.67 | 45.55 | 0.06 | Tchem |
| SIGMAR1 | 88.94 | 51.77 | 0.06 | Tclin |
| HTT | 63.23 | 49.76 | 0.06 | Tchem |
| TRAM1 | 26.98 | 26.55 | 0.06 | Tdark |
| SFXN4 | 26.68 | 25.86 | 0.06 | Tdark |
| DHRS7B | 23.98 | 22.85 | 0.06 | Tdark |
| CDK15 | 25.66 | 26.08 | 0.06 | Tchem |
| GPR21 | 8.58 | 19.63 | 0.06 | Tdark |
| ALPK2 | 23.51 | 22.99 | 0.06 | Tbio |
| LTK | 32.66 | 42.36 | 0.06 | Tclin |

**IDG Generated Resources**

As the IDGE program has moved into an implementation phase, members of the IDG have begun generating new data or reagents. These resources are focused on three main target families, GPCRs, ion channels and kinases. The IDG reagents include the following types: antibody, cell, genetic construct, mouse, peptide, and small molecule while the IDG data cover: affinity purification mass spectrometry data, cyclic immunofluorescence data, expression data, immunohistochemistry data, channel activity data, KINOMEscan data, mouse phenotype data, NanoBRET data, proteomics data, probe, chemical tool, and GPCR mouse imaging data. The IDG resources are deposited and IDG Consortium pre-agreed repositories (as listed in <https://druggablegenome.net/PublicRepositories>) and they are formally described by a set of metadata standard specifications (<https://druggablegenome.net/MetadataStandards>) and descriptors used to represent them in Pharos.

Table S2**.** List of the Pharos IDG resource descriptors. The IDG resources categorized into two main categories, reagents and data, are represented by a set of descriptors shown in the table. While all IDG reagents (antibody, cell, genetic construct, mouse, peptide and small molecule ) share some common descriptors shown under ‘Reagent’ category, each of these reagent categories also have additional category-specific descriptors, labeled with (*) in the table. In a similar way, the common data elements describing all data types (Affinity purification mass spectrometry data, Cyclic immunofluorescence data, Expression data, Immunohistochemistry data, Channel activity data, KINOMEscan data, Mouse phenotype data, NanoBRET data, Proteomics data, Probe, Chemical tool, and GPCR Mouse Imaging data) are listed under the ‘Data’ category while probe, chemical tool, and GPCR mouse imaging data have a few additional specifications, labeled with ** in the table. These descriptors are used to represent the IDG resources in Pharos.

| Resource type | Specification | Description |
| --- | --- | --- |
| Reagent | Name | Reference name of reagent |
| Reagent | Repository | Physical repository for the IDG resource |
| Reagent | Repository page link | External link to physical repositories |
| Reagent | Data page link | External link to relevant published data |
| Antibody* | Usage | Experiments and assays for which the antibodies have been tested and validated |
| Antibody* | Antibody ID | Identifier as registered by a repository |
| Cell* | Type | Cell type - primary, iPS, xenograft, etc. |
| Genetic Construct* | Vector type | Purpose/type of construct |
| Mouse* | MMRRC_ID | ID as registered with MMRRC |
| Mouse* | Corresponding construct | Link to the external repository where the construct was registered |
| Mouse* | Allele | Specific nature of genetic modification |
| Peptide* | PRM type | If peptide used in PRM-based experiments |
| Small Molecule* | Canonical SMILES | Canonical SMILES sequence of molecule |
| Small Molecule* | External ID registration system | Repository corresponding to the external ID |
| Small Molecule* | External ID | External ID (PubChem, ChEBI, ZINC, etc.) |
| Data | Title | Name/title of the dataset |
| Data | Authors | Person(s) who performed corresponding experiments |
| Data | Data Link | Link to external data repository containing key dataset metadata |
| Probe, Chemical tool** | Name | Canonical (trade or IUPAC) name of molecule |
| Probe, Chemical tool** | Canonical SMILES | Canonical SMILES of molecule |
| Probe, Chemical tool** | Ligand type | Mode of action towards given gene |
| Probe, Chemical tool** | External ID | External ID (PubChem, CheBI, ZINC, etc.) |
| Probe, Chemical tool** | External registration system | Repository corresponding to the external ID |
| Probe, Chemical tool** | Activity | On target activity |
| Probe, Chemical tool** | Selectivity | Selectivity against similar genes |
| Probe, Chemical tool** | Repository page link | Link to the vendor for the physical sample |
| Probe, Chemical tool** | Link to the supporting data | External link to the data |
| Probe** | Probe page | Link to chemicalprobes.org where the probe is registered |
| Probe** | Negative control | Name of inactive analog |
| Probe** | Negative control repository page | Link to the vendor for the physical sample of negative control |
| Probe** | Negative control Canonical SMILES | Canonical SMILES of inactive analog |
| GPCR Mouse Imaging** | Name | Mouse name as registered with repository |
| GPCR Mouse Imaging** | MMRRC_ID | ID as registered with MMRRC |
| GPCR Mouse Imaging** | Repository page link | Link to the vendor for the physical sample |
| GPCR Mouse Imaging** | Sex | Describes sex of the screened mouse |
| GPCR Mouse Imaging** | Expression data | Is the gene expressed or not? |
| GPCR Mouse Imaging** | Tissue | Standardized name (from UBERON) |
| GPCR Mouse Imaging** | Tissue ID | UBERON ID |
| GPCR Mouse Imaging** | Data page link | Link to the corresponding preselected images to support the conclusion |
